# Hearing the shape of a drum for light: isospectrality in photonics

**DOI:** 10.1515/nanoph-2021-0614

**Published:** 2021-12-08

**Authors:** Seungkyun Park, Ikbeom Lee, Jungmin Kim, Namkyoo Park, Sunkyu Yu

**Affiliations:** Photonic Systems Laboratory, Department of Electrical and Computer Engineering, Seoul National University, Seoul 08826, Korea; Intelligent Wave Systems Laboratory, Department of Electrical and Computer Engineering, Seoul National University, Seoul 08826, Korea

**Keywords:** Darboux transformation, dimensionality, Householder transformation, isospectrality, Lanczos transformation, supersymmetry

## Abstract

The independent tailoring of wave quantities lays the foundation for controlling wave phenomena and designing wave devices. The concept of isospectrality, which suggests the existence of systems that provide identical spectra, has inspired a novel route to the spectrum-preserved engineering of wave–matter interactions in photonics, acoustics, and quantum mechanics. Recently, in photonics, constructing isospectral optical structures has become an emerging research topic to handle the intricate spectral responses of the systems composed of many-particles or inhomogeneous materials. The cornerstones in this field have stimulated the realization of non-Hermitian systems with real eigenspectra, one-dimensional structures exhibiting higher-dimensional physics, and novel engineering methodologies for broadband devices such as phase-matched multiplexers and multimodal lasing platforms. Here we review recent achievements based on isospectrality in photonics. We outline milestones in two different subfields of supersymmetric photonics and interdimensional isospectrality. We illustrate that isospectrality has paved the way for the independent control of wave quantities, showing great potential for the analytical and platform-transparent design of photonic systems with complex structures and materials.

## 1 Introduction

A light wave is characterized by multiple physical quantities defined in spatial and temporal domains, their reciprocal spaces, and the other intrinsic spaces of photons such as polarization [1]. Optical phenomena are then described by the evolutions and interactions of those physical quantities. For example, refraction or reflection corresponds to the alteration of wavevectors, and light–matter interactions in anisotropic or structured materials can lead to polarization rotations [2] or spin–orbit interactions [3]. Information processing through light waves then requires handling such physical quantities in the desired manner, especially independent control of the target physical quantities.

However, such independent handling of optical quantities is usually a challenging issue due to their mutual connections, which are generally described with nonlinear functions of a large number of system parameters even in a straightforward environment. For example, consider the eigenfrequencies of a whispering-gallery-mode resonator. These physical quantities are determined by multiple parameters, such as the structural boundaries [4] and material compositions [5]. Although the engineering of the resonator possesses almost infinite design freedom in the boundary deformation or refractive index profiles, the change of a system parameter affects all the eigenmodes nonlinearly through Maxwell’s equations, hindering the independent control of the target eigenfrequency. Therefore, the development of an analytical and platform-transparent method for the independent engineering of optical quantities, which should be distinct from brute-force numerical methods, is strongly desired in both fundamental and applied research in photonics.

As an example of the independent control of wave quantities, in this review, we introduce the emerging field in photonics, which originates from the concept of isospectrality. Starting from the comprehensible description of isospectrality, we focus on two subfields: supersymmetric photonics and interdimensional isospectrality, covering analytical methods such as Darboux, Householder, and Lanczos transformations, extraordinary phenomena in phase matching, non-Hermitian potentials, and higher-dimensional physics, and device applications such as multimodal filtering and broadband switching. For future research, we also briefly discuss the related field of hyperuniformity and machine learning, which corresponds to the realization of quasi-isospectrality with statistical or numerical tools.

## 2 Isospectrality in photonics

**Figure 1:**
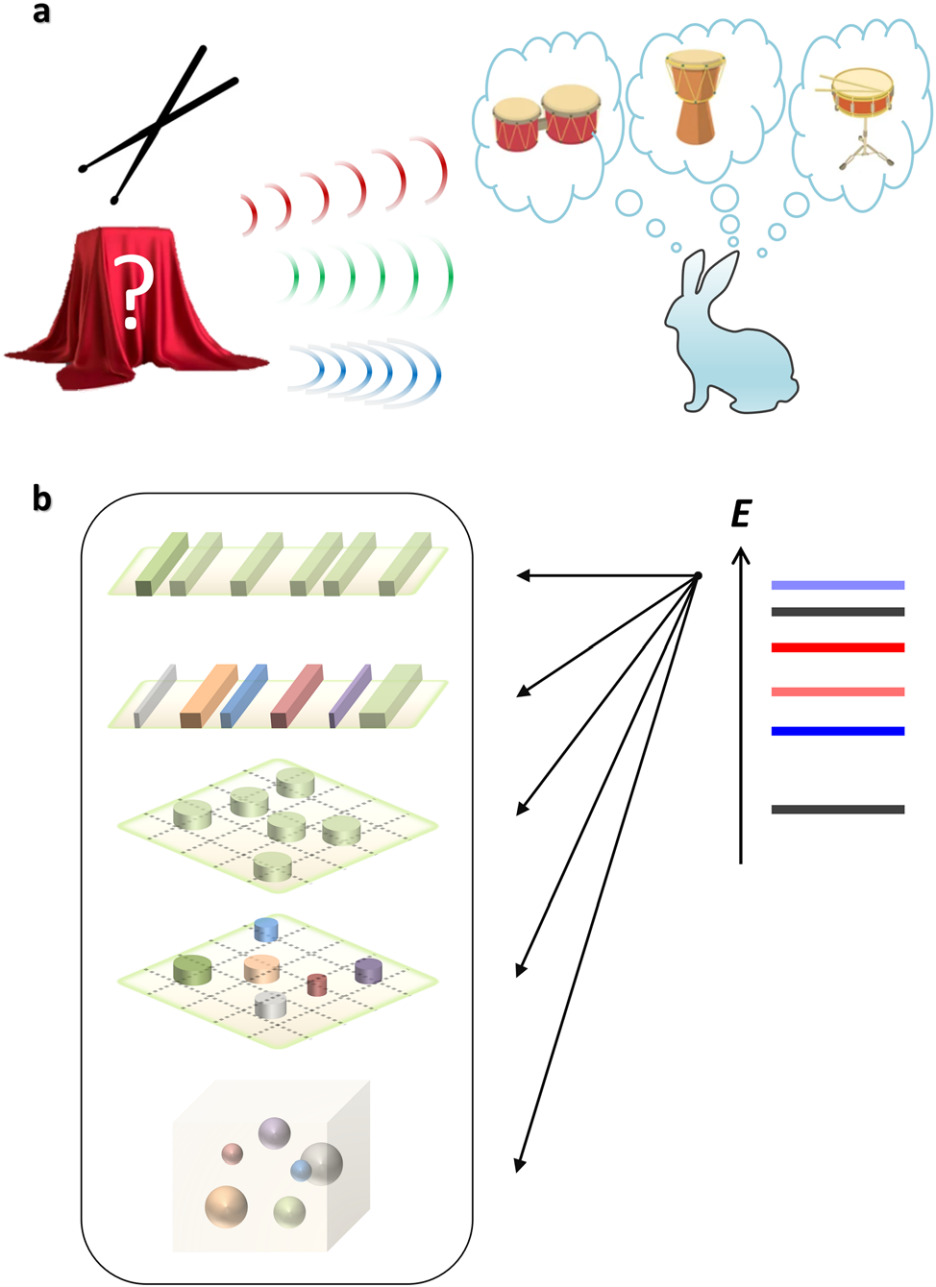
Concept of isospectrality. (a) Can this rabbit describe the shape of a drum behind a veil by hearing only its sound spectrum? It is impossible because there can exist different shapes of drums that provide the same sound spectrum. Such a family of drums satisfies “isospectrality”. Image credits: a veiled drum (Viktoriya_Y/Shutterstock.com), drum sticks (martialred/Shutterstock.com), drums (ViktoriaKazakova/Shutterstock.com). (b) In wave mechanics, we can also expect a family of materials or structures having different compositions or dimensionalities, which provide the same (real or complex) eigenspectrum *E*.

First, imagine a drum behind a veil. When one plays the drum and you hear enough of its sound, can you guess the shape of the drum without seeing it? This intriguing situation describes the underlying concept of Kac’s classic question of “Can one hear the shape of a drum?” [6], which implied the concept of isospectrality. The sound spectrum of the drum is composed of the superposition of pure tones. Therefore, to successfully predict the shape of the drum, a unique drum shape should exist for a given set of generated pure tones (Figure 1a).

Mathematically, in Kac’s original paper [6], the vibration of the membrane in the drum is described by the Helmholtz equation with the Dirichlet boundary condition:

(1) $$\frac{1}{2}\nabla^{2}U+\omega^{2}U=0\text{ in }\Omega_{1},\;U=0\text{ on }\Gamma_{1}\text{.}$$

where *U* represents the amplitude of the membrane vibration at the frequency *ω*, and Γ_1_ denotes the boundary of the membrane Ω_1_. Then, M. Kac’s question asks whether the other membrane that satisfies the following relationship exists

(2) $$\frac{1}{2}\nabla^{2}V+\omega^{2}V=0\text{ in }\Omega_{2},\;V=0\text{ on }\Gamma_{2}\text{.}$$

where *V* represents the amplitude of the membrane vibration at the frequency *ω*, and Γ_2_ denotes the boundary of the membrane Ω_2_. If there is no other membrane satisfying Eq. (2) with Ω_2_ ≠ Ω_1_, there exists only a unique shape of a membrane, Ω_1_, for a given set of eigenfrequencies *ω*. Because of this uniqueness, one may predict (or hear) the shape of a drum for a set of given tones. Otherwise, without any contradiction to the sound spectrum, one can imagine different shapes of a membrane generating the same sound spectrum, satisfying “iso”-spectrality. Notably, this classic problem was solved in 1992 in a seminal paper demonstrating that “one cannot hear the shape of a drum” [7]. This work successfully demonstrates a one-to-many relationship between a set of pure tones and the corresponding membrane shapes, eventually forming isospectrality (Figure 1a).

The concept of isospectrality [8], stating the one-to-many relationship between the spectral responses and the systems, has been generalized into many different fields, including network science [9], fractal physics [10], and graph theory [11]. In particular, the field of wave mechanics, possessing various eigenvalue problems, provides a suitable platform for examining and exploiting the concept of isospectrality. In photonics, the field of isospectrality allows for the design of optical structures or devices that possess identical spectral responses (Figure 1b), for example, a family of nanostructures that generate the same color spectrum. The necessity of isospectrality in photonics is also the independent engineering of wave quantities: altering the selected wave quantities (e.g., momentum, wavefront, localization and transport, or topology) while preserving the spectral response.

In photonics, or more generally, classical and quantum wave mechanics, wave behaviors inside a system can be described by the Hamiltonian *H*, which includes the optical potential of the system. Then, for stationary states, the governing equation of the system is the Hamiltonian equation *Hψ*_*E*_ = *Eψ*_*E*_, where *E* is the eigenvalue and *ψ*_*E*_ is the corresponding eigenmode. The basic strategy for achieving isospectrality is the application of operator *A* to the original Hamiltonian equation *AHψ*_*E*_ = *AEψ*_*E*_ = *EAψ*_*E*_. When the relation such as *AH* = *H*_iso_ *A*, which is called the “intertwining relation” [12], is satisfied, we can rewrite the equation as *H*_iso_(*Aψ*_*E*_) = *E*(*Aψ*_*E*_), maintaining the form of the eigenvalue problem. The newly obtained equation from operator *A* then has the eigenvalues *E* and the corresponding eigenvectors *Aψ*_*E*_. Therefore, the condition of isospectrality in wave mechanics is to find the proper operator *A* that guarantees the physically allowed form of *H*_iso_ for the relation *AH* = *H*_iso_ *A*, whether the original Hamiltonian *H* is Hermitian or non-Hermitian.

Many fundamental operators, such as translation, rotation, and parity conversion, satisfy the above criteria for isospectrality. However, these simple operators lead to trivial solutions, effectively the same structure as the original one, just having translation, rotation, or mirror reflection. More interesting and useful results for theoretical and engineering achievements require nontrivial operators *A*, resulting in fundamentally different systems from the original one while achieving isospectrality. In the following sections, we focus on two emerging analytical tools for nontrivial isospectral transformations: supersymmetric transformation and interdimensional isospectrality.

## 3 Isospectrality in supersymmetric photonics

Supersymmetry (SUSY) is an intriguing conjecture that implies a hidden connection between integer spin bosons and half-integer spin fermions in particle physics [13, 14]. SUSY is one of the critical subtheories in string theory [15], suggesting the existence of a pair of elementary particles having the same mass and quantum number except for different spin numbers [13]. Although the validity of SUSY and string theory is still in question due to the lack of experimental evidence [16], the mathematical technique used in SUSY particle physics provides a novel toolkit for handling quantum-mechanical problems, opening a new field of supersymmetric quantum mechanics (SUSY QM) [17].

The basic mathematical tool in SUSY QM is operator factorization [18], which attempts to decompose the original Hamiltonian *H*_o_ into two operators as *H*_o_ = *A* ^†^ *A*, where *A* ^†^ is the Hermitian adjoint of the partial operator *A*. When treating *A* ^†^ and *A* as the generalization of the creation and annihilation operators in quantum harmonic oscillators, we can introduce the reversal of the order of two operators, constituting the SUSY partner Hamiltonian *H*_s_ = *AA* ^†^. Therefore, in addition to the finding of a mathematically accessible factorization *H*_o_ = *A* ^†^ *A*, SUSY QM requires the existence of a physically allowed form of *H*_s_ = *AA* ^†^, providing the SUSY partner relationship between the original potential *V*_o_ of *H*_o_ and the SUSY partner potential *V*_s_ of *H*_s_. Notably, it is also known that the mathematical technique applied to the factorization in SUSY QM, which is the Darboux transformation [19], provides an analytically solvable and physically allowed form of *H*_s_ = *AA* ^†^ for one-dimensional (1D) potential problems [17]. In terms of reproducing such a SUSY QM theory in photonics [20, 21], the natural starting point is then to find the description of photonic systems mathematically similar to that of quantum mechanics.

### 3.1 Quantum-optical analogy

In physics, an analogy is a powerful tool to connect phenomena in various fields, which are connected through the same form of mathematical descriptions. The similarity between the Schrödinger equation and Maxwell wave equation has enabled quantum-optical analogy [22], which enables the demonstration of ∼Å scale quantum phenomena in μm-mm scale optical platforms. We note that quantum-optical analogy has been studied in different platforms and physical axes with distinct mathematical formulations, ranging from continuous to discrete systems in spatial or temporal domains. As an example, consider the analogy of temporal quantum-mechanical phenomena with spatial optical phenomena in continuous systems. In a slowly varying envelope approximation, light propagating along the *z*-axis in nonmagnetic optical materials is described by the following paraxial wave equation for an amplitude of an electric field *ψ*:

(3) $$i\frac{1}{k_{0}}\frac{\partial \psi }{\partial z}=-\;\frac{1}{2k_{0}^{2}n_{0}}\nabla_{\text{T}}^{2}\psi -\Delta n\left(x,y\right)\psi \text{,}$$

where *k*_0_ is the free-space wavenumber, *n*_0_ is the average refractive index, ∇_T_^2^ is the transverse Laplacian operator, and Δ*n* is the profile of the refractive index perturbation. This equation has the same mathematical form as the Schrödinger equation with *λ*_0_/2*π* = 1/*k*_0_ ∼ *ħ* = *h*/2*π*, *n*_0_ ∼ *m*, −Δ*n* ∼ *V*, and *z* ∼ *t*, emphasizing the analogy between a refractive index profile Δ*n*(*x*,*y*) and a quantum potential. The evolution of an electric field along the *z*-axis then corresponds to the optical analogy of a time-varying quantum wavefunction. The well-developed fabrication of weakly perturbed Δ*n*(*x*,*y*) has enabled the classical observation of QM phenomena that are significantly difficult to measure in QM systems, such as Bloch oscillations [23, 24], Zener tunneling [25, 26], Rabi oscillations [27], adiabatic passage [28], and Fano resonances [29, 30]. We note that quantum-optical analogy can also be achieved in discrete systems composed of weakly-coupled wave elements, which allows the modeling of quantum-mechanical tight-binding structures with spatial or temporal evolutions of classical light in optical waveguides [31] or resonators [32], respectively. Numerical techniques and achievements in quantum-optical analogy can also be applied to the analogy of quantum phenomena with other types of wave systems, such as acoustic [33] or elastic waves [34].

### 3.2 Theoretical foundation

In the field of quantum-optical analogy, the results on SUSY QM can be successfully reproduced in optical platforms [20, 35]. Because the mathematical tool in SUSY QM allows for only the handling of 1D potentials [17], we consider the transverse electric (TE) mode *E*_*y*_(*x*,*z*) = *ψ*(*x*)e^i*βz*^ propagating on the *z*–*x* plane with the propagation constant *β*. The original optical Hamiltonian *H*_o_ then has the form of

(4) $$H_{\text{o}}=-\;\frac{d^{2}}{dx^{2}}-k_{0}^{2}n_{\text{o}}^{2}\left(x\right)\text{.}$$

where *n*_o_(*x*) is an original refractive index profile. Light propagations in this optical potential are then described with the eigenvalue problem *H*_o_ *ψ*_o,*m*_ = *γ*_*m*_ *ψ*_o,*m*_, where *γ*_*m*_ = −*β*_*m*_^2^ is the *m*th eigenvalue and *ψ*_o,*m*_ is the *m*th eigenmode. Considering a nonzero ground state eigenvalue *γ*_0_ in optical problems, the factorization for SUSY photonics is established as follows:

(5) $$H_{\text{o}}=A^{†}A+\gamma_{0}\text{ and }H_{\text{s}}=AA^{†}+\gamma_{0}\text{,}$$

where *H*_s_ is the SUSY partner Hamiltonian of *H*_o_, *A* = +*d*/*dx* + *W*(*x*), *A* ^†^ = −*d*/*dx* + *W*(*x*), and *W*(*x*) is the superpotential, which is the solution of the Riccati equation [36]. In terms of the solution of the Riccati equation, we can achieve the particular solution of *W*(*x*) according to the ground state solution of the eigenvalue problem in Eq. (6), as

(6) $$W\left(x\right)=-\;\frac{d}{dx}\text{ln}\left(\psi_{\text{o,}0}\left(x\right)\right)\text{.}$$

This particular solution of *W*(*x*) corresponds to unbroken SUSY, while the other solutions represent broken SUSY [17, 35]. For both cases, we obtain the connection between the refractive index profiles of *H*_o_ and *H*_s_ as follows:

(7) $$n_{\text{s}}\left(x\right)=\sqrt{{\left(n_{\text{o}}\left(x\right)\right)}^{2}-\frac{2}{k_{0}^{2}}\frac{d}{dx}W\left(x\right)}\text{.}$$

**Figure 2:**
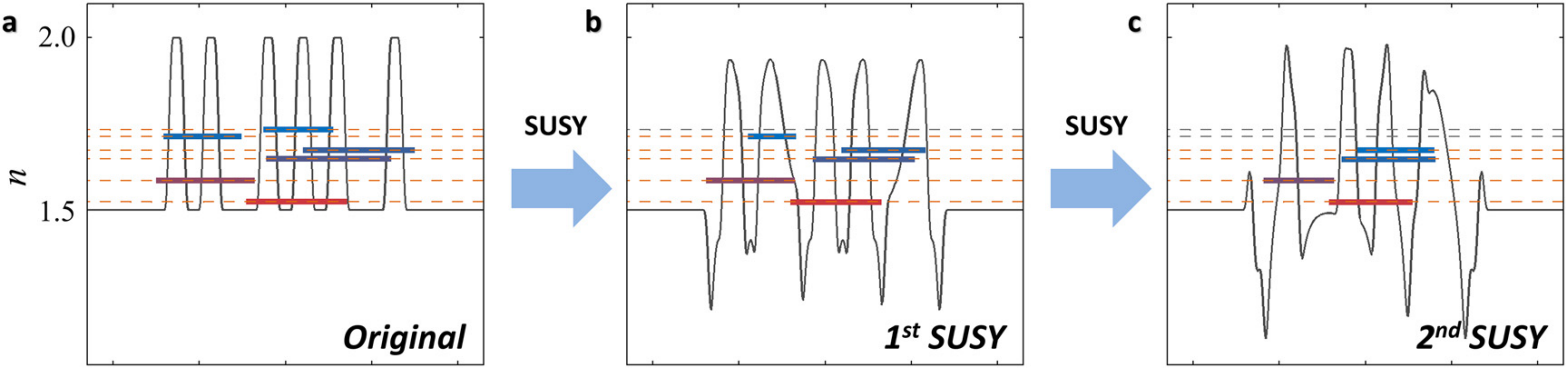
SUSY transformation for quasi-isospectral materials. (a–c) An example of multiple SUSY transformations. (a) Original potential. (b) The 1st and (c) 2nd SUSY-transformed potentials. The *n*-axis represents both the refractive index profile and effective index of optical modes. The orange (or black) dotted lines denote the preserved (or annihilated) eigenstates. Note that because of the different signs in optical and quantum potentials of −*n* ∼ *V*, the ground state has the largest value of *n*. Panels (a–c) are adapted from [37], CC BY 4.0 (https://creativecommons/org/licenses/by/4.0/).

Due to the mathematical similarity between classical optics and quantum mechanics, SUSY photonics inherits the intriguing properties of SUSY QM. First, from *H*_o_ *ψ*_o,*m*_ = (*A* ^†^ *A* + *γ*_0_)*ψ*_o,*m*_ = *γ*_*m*_ *ψ*_o,*m*_, we achieve *AH*_o_ *ψ*_o,*m*_ = *A*(*A* ^†^ *A* + *γ*_0_)*ψ*_o,*m*_ = (*AA* ^†^ + *γ*_0_)(*Aψ*_o,*m*_) = *γ*_*m*_(*Aψ*_o,*m*_), constructing the isospectrality of *H*_s_(*Aψ*_o,*m*_) = *γ*_*m*_(*Aψ*_o,*m*_) with eigenmodal transformation *Aψ*_o,*m*_ (Figure 2). It is noted that this SUSY transformation satisfies the intertwining relation *AH* = *H*_iso_ *A* in the form of *AH*_o_ = *H*_s_ *A*. More interestingly, when we use the superpotential *W*(*x*) for unbroken SUSY, the transformation leads to the annihilation of the original ground state *Aψ*_o,*m*_ = *O* [17], resulting in the shift of the ground-state eigenvalue in the SUSY-transformed Hamiltonian (Figure 2). Furthermore, as shown in the deformation of the refractive index profile due to *W*(*x*) in Eq. (7), the spatial profile of the newly obtained ground state of the SUSY Hamiltonian is generally different from that of the original Hamiltonian. These ground-state perturbations in terms of the eigenvalue shift and profile deformation of the SUSY Hamiltonian then allow for the series of SUSY transformations (Figure 2), which leads to a family of quasi-isospectral potentials with different landscapes [17, 20, 35]. This result satisfies the necessary conditions of various photonic devices. It is also worth noting that the operator factorization for the Hamiltonian with a continuous potential profile can be substituted with other numerical factorizations for the SUSY transformation of discrete Hamiltonians, such as the application of Cholesky [20, 37] or QR [38] factorizations to coupled mode theory [39].

### 3.3 1D applications

**Figure 3:**
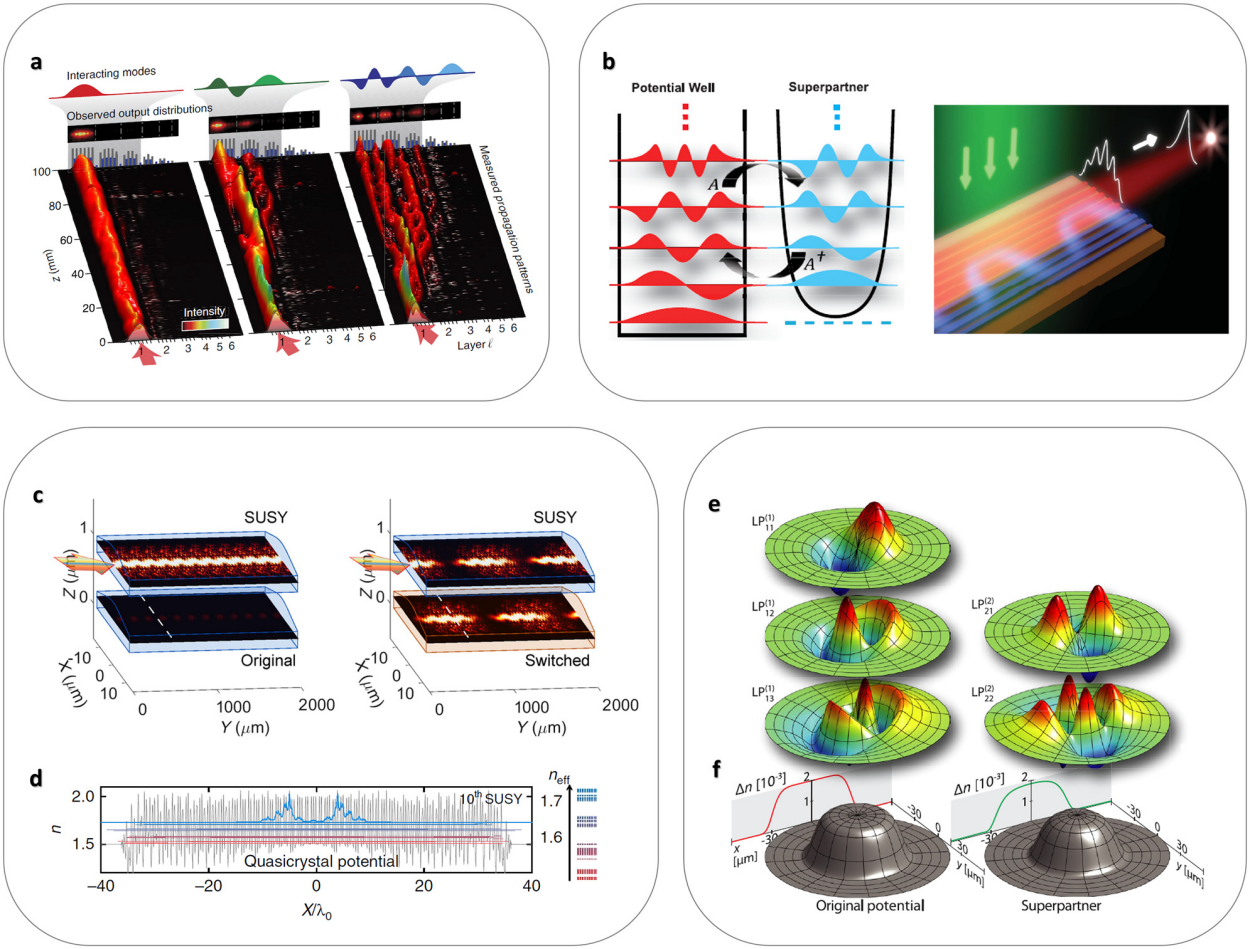
1D SUSY photonics and its applications. (a) Mode-division multiplexer based on mode separation in a SUSY ladder [21]. Light injected into the ground state of the original potential remains trapped without coupling into SUSY partners. (b) SUSY laser array composed of an original lasing lattice (red) and its coupled and lossy SUSY partner (blue) [41]. The SUSY laser emits in the ground-state (in-phase) mode of the original lasing part. (c) Binary random wave switching in the SUSY harmonic pair: (left) off and (right) on states [43]. The input wavefront is fully reconstructed at the full coupling position in the output [dotted lines in (c)]. (d) Disordered bandgap material obtained from the SUSY-transformed quasicrystal: 10 successive SUSY transformations [37]. The blue curve represents the ground state, and *n*_eff_ denotes the eigenspectrum. (e) SUSY bound states from (f) the SUSY-transformed cylindrically symmetric index profiles of azimuthal numbers *ℓ*_1_ = 1 and *ℓ*_2_ = 2 [20]. Panel (a) is adapted from [21], CC BY 4.0 (https://creativecommons/org/licenses/by/4.0/). Panel (b) is adapted with permission from [41], ⓒ AAAS. Panel (c) is adapted with permission from [43], ⓒ APS. Panel (d) is adapted from [37], CC BY 4.0 (https://creativecommons/org/licenses/by/4.0/). Panels (e and f) are adapted with permission from [20], ⓒ APS.

Due to the original mathematical form of the SUSY transformation described above—1D transformation for Hermitian Hamiltonians—various important applications using the SUSY transformation have focused on the engineering of 1D optical potentials without gain or loss. First, the SUSY transformation provides the global phase matching condition between weakly coupled original and SUSY potentials except for the original ground state [20], realizing the independent control of the optical ground state. By employing a series of SUSY transformations to a 1D multimode optical waveguide or coupled waveguides, one can achieve a family of platforms that allow for independent access to individual eigenmodes [20, 37]. This unique property has been applied to the construction of a mode-division multiplexing (MDM) device for integrated photonics, which consists of SUSY partner waveguides [21] (Figure 3a). Due to the global phase matching condition first demonstrated in a previous study [20], the proposed MDM platform enables broad bandwidth mode conversion with high fidelity [21]. Based on the first theoretical proposal on supersymmetric laser arrays [40], the SUSY transformation has also been employed for modal filtering in multimodal lasing devices, which is achieved with a dissipating SUSY partner applied to only a part of the eigenmodes (Figure 3b) [41, 42]. A similar technique has also been applied to digital switching devices for multimode operation, achieving complete random wave switching (Figure 3c) and fuzzy logic elements using the SUSY transformation of harmonic and Pöschl–Teller potentials, respectively [43].

The quasi-isospectral feature of the SUSY transformation has also enabled the independent control of other wave quantities beyond spectral responses in 1D systems, such as bandgaps [37, 44] and localization properties [37]. First, due to the deformation of potential profiles, the profile of eigenmodes according to the transformation of *Aψ*_o,*m*_ leads to the SUSY-enabled isospectral control of wave localization inside a material [37]. The cascaded application of SUSY transformations to crystals or quasicrystals allows for disordered bandgap materials (Figure 3d). By controlling the number of SUSY transformations, the statistical transition of potential landscapes from negative to zero and to positive correlations can be achieved, and the SUSY deformation can lead to significant wave localization inside SUSY-transformed potentials. In terms of engineered disorder [45], this result breaks the traditional relationship between spectral bandgaps and modal localization by devising SUSY-transformed structures supporting wave localization while preserving the bandgap of an original crystal or quasicrystal. The SUSY transformation can also be applied to other 1D problems, such as the conservation of scattering properties for any angle of incidence [20, 46], the control of orbital angular momenta in SUSY-transformed structures with rotational symmetry (Figure 3e and f) [20, 47], and the topological transition using the QR-factorized SUSY transformation [38].

### 3.4 Generalization

To lift several restrictions on traditional SUSY transformations—1D Hermitian transformations for spatial-domain governing equations—various cornerstones have been demonstrated recently. First, by generalizing the factorization *H*_o_ = *A* ^†^ *A* + *γ*_0_ to *H*_o_ = *BA* + *γ*_0_, where *B* ≠ *A* ^†^, SUSY transformations can be applied to non-Hermitian systems [48, 49]. From the relation *AH*_o_ *ψ*_o,*m*_ = *A*(*BA* + *γ*_0_)*ψ*_o,*m*_ = (*AB* + *γ*_0_)(*Aψ*_o,*m*_) = *γ*_*m*_(*Aψ*_o,*m*_), the SUSY-transformed Hamiltonian *H*_s_ = *AB*, which is non-Hermitian, also satisfies the isospectrality with the transformed eigenmodes *Aψ*_o,*m*_. Although such a non-Hermitian Hamiltonian can be constructed in open quantum systems [50], an optical system composed of gain and loss materials is one of the most proper platforms for demonstrating non-Hermitian Hamiltonians [50], [51], [52].

**Figure 4:**
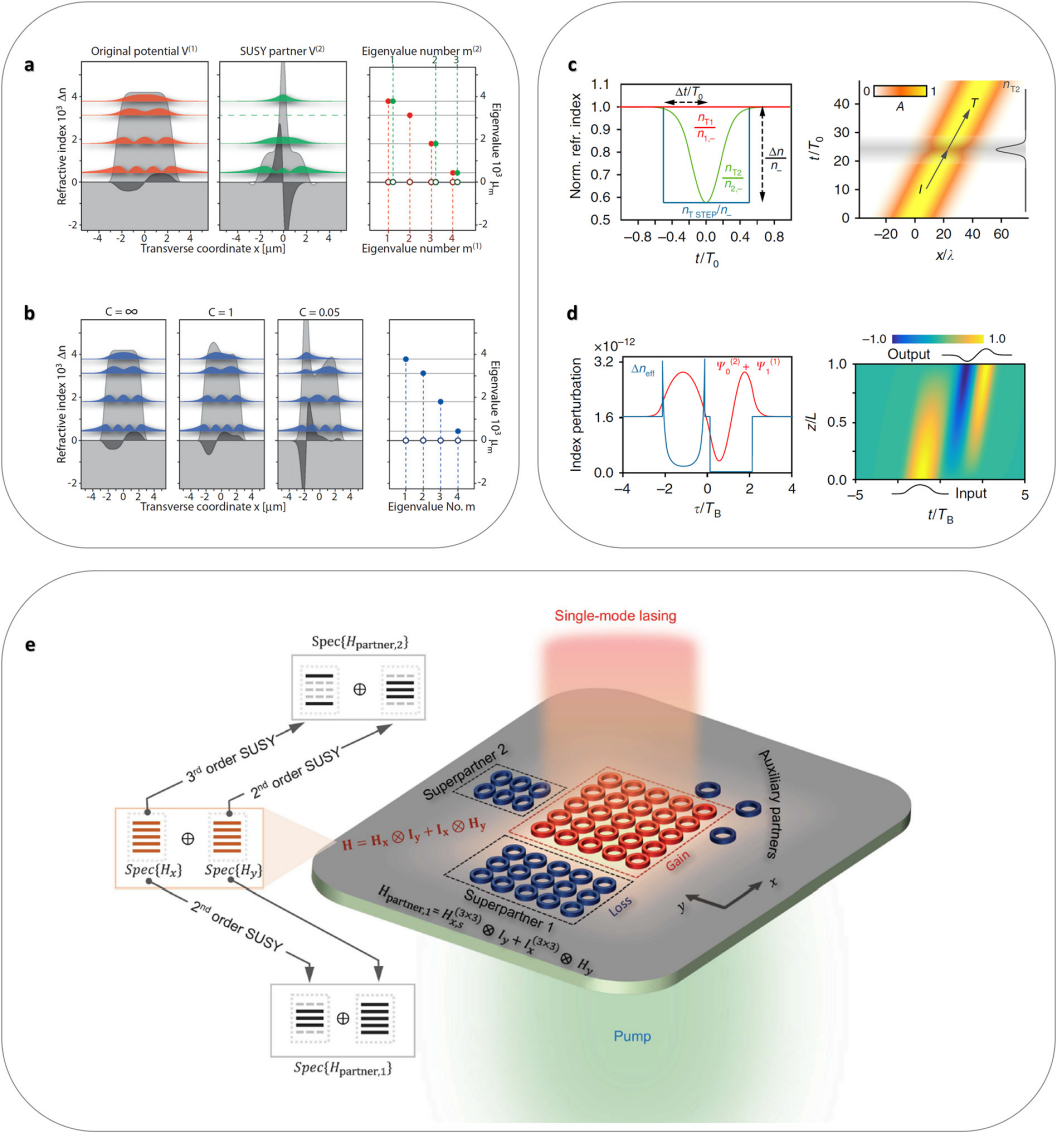
Generalized SUSY photonics. (a and b) Non-Hermitian SUSY transformations [48]. (a) Excited-state annihilation [48]. For a PT-symmetric multimode waveguide (orange: bound states), the SUSY transformation removes the second mode (green). Right figure: the eigenvalue spectra of the structures. (b) Non-PT-symmetric conservative potentials [48]. By controlling an arbitrary integration constant C for the general solution of the Riccati equation, non-PT-symmetric SUSY partners with real eigenspectra are achieved. Right figure: identical eigenvalue spectrum of the structures. (c and d) Temporal SUSY transformations [60]. (c) Reflectionless time-varying material [60]. Red, green, and blue lines denote the constant potential, its SUSY partner, and square well. Δ*t*/*T*_0_ and Δ*n*/*n*_–_ are obtained from the temporal SUSY transformation for the finite range, where *T*_0_ = 2π/*ω*_0_ and *ω*_0_ is the center frequency. Right figure: scattering-free propagation in the SUSY partner (green line in the left figure). (d) Pulse-shape transformer [60]. Blue and red lines denote the time-varying potential Δ*n*_eff_ and overlapping eigenmodes *Ψ*_0_^(2)^ + *Ψ*_1_^(1)^, where *Ψ*_0_^(2)^ (or *Ψ*_1_^(1)^) is the first-excited (or ground) state in the right (or left) potential. The left potential is the SUSY partner of the right potential. The right figure shows the modal transformation along the temporal axis. (e) Higher-dimensional SUSY microlaser array [65], composed of an original gain system (red array) and its higher-order (2nd- and 3rd-order) SUSY partners (blue arrays). Decomposing the entire spectrum to a Kronecker sum allows the 1D SUSY transformation to each axis. Three auxiliary lossy elements are included for the complete dissipation of excited states. Panels (a and b) are adapted with permission from [48], ⓒ APS. Panels (c and d) are adapted from [60], CC BY 4.0 (https://creativecommons/org/licenses/by/4.0/). Panel (e) is adapted with permission from [65], ⓒ AAAS.

This non-Hermitian SUSY transformation has unique features when compared with a Hermitian transformation (Figure 4a and b). First, due to the lack of the eigenmode node (*ψ*_o,*m*_ = *O*) in general non-Hermitian potentials, the SUSY transformation can be developed not only with the ground-state gauge *BA* + *γ*_0_ but also with the excited-state gauge *BA* + *γ*_*m*≠0_. Therefore, the annihilation of higher-order states is available in non-Hermitian systems, allowing for higher-order mode selective devices (Figure 4a). Second, when we utilize the broken SUSY transformation through a general solution of the Riccati equation [36], which leads to broken spatial symmetry in the resulting potential profile [35], a parity-time- (PT) symmetric potential is transformed into a non-PT-symmetric potential while completely preserving the spectrum of the PT-symmetric potential (Figure 4b). This allows for the construction of complex potentials having real eigenspectra without PT symmetry, even achieving highly disordered complex potentials with pseudo-Hermiticity [48]. In a similar context, the realizations of resonant propagations in supersymmetric parametric oscillators [49], higher-order exceptional points [53], and one-way reflectionless PT-symmetric crystals [54] have also been studied with non-Hermitian SUSY transformations.

Due to the similarity between the wave equations described in the spatial and temporal domains, intriguing phenomena shown in the 1D spatial system have been intensively reproduced in the temporal system, such as the temporal analogy of refraction and reflection [55], temporal crystals [56] and disordered structures [57], and dynamical topology [58, 59]. In these temporal systems, the continuous translational symmetry in time is broken, resulting in the alteration of energy while preserving momentum. Another crucial difference is the causality along the temporal axis, permitting only the unidirectional propagation of light along the +*t*-axis. With these key features of temporal systems, the application of the SUSY transformation to spatial profiles of the refractive index has been reproduced in temporal responses, enabling various intriguing applications from isospectrality [60] (Figure 4c and d). For example, the SUSY partner of a constant refractive index, having time-varying modulation, allows reflectionless, all-dielectric, and frequency-selective transparent windows, which are independent of polarization and propagation direction (Figure 4c). The series of original and SUSY partner temporal waveguides also enables the construction of a reconfigurable pulse-shape transformer (Figure 4d), which is the temporal counterpart of spatial MDMs [21]. Although various other applications, such as perfect phase shifters and optical isolators, were also suggested in [60], the limitations of temporal SUSY devices are the necessity of large and rapid variations in refractive indices, which can be overcome with epsilon-near-zero media [61] or phase change materials [62].

Another hurdle in SUSY transformations is its mathematical and physical validity restricted to 1D systems. The most straightforward but incomplete method for the multidimensional extension of SUSY transformations is the separation of variables [34, 37, 63]. For example, when the 2D refractive index profile *n*(*x*,*y*) satisfies the form *n* ^2^(*x*,*y*) = *n* ^2^(*x*) + *n* ^2^(*y*), the 2D Hamiltonian can be separated into two independent 1D Hamiltonians. When applying the SUSY transformation to each 1D partial Hamiltonian, annihilation of the excited states in the 2D Hamiltonian can also be achieved [37]. This separation-of-variables technique has recently been advanced to the SUSY handling of coupled polarization problems in elastic waves [34], the sharing of a subset of an eigenspectrum in discrete SUSY systems [64], and the 2D tight-binding Hamiltonian that can be decomposed into the Kronecker sum of 1D Hamiltonians (Figure 4e) [65]. According to the Kronecker sum relationships on the eigenspectra of each Hamiltonian, the 2D SUSY partner can be constructed by realizing higher-order SUSY partners for each 1D Hamiltonian. This work allows precise mode manipulation in microlaser arrays for single-mode lasing by developing a platform composed of a gain resonator array and its lossy SUSY partners with state annihilations. Notably, because of additional state annihilations during higher-order SUSY transformations, some auxiliary elements are used in [65]. Other mathematical techniques, such as time-dependent Darboux transformation [66], Moutard transformation [67], or Cholesky decomposition for a specific form of Hamiltonians [68], have also been intensively studied to handle multidimensional systems.

## 4 Isospectrality in dimensionality engineering

The dimensionality of geometry is a critical factor in understanding and manipulating physical phenomena. In photonics, an illustrative example is found in the geometry of optical elements: 0D dots, 1D waveguides, 2D layers, and 3D bulk media. Electromagnetic responses from these elements are described by mathematically different forms of Green’s functions [69], which are the origin of distinct bound and unbound states, scattering profiles and efficiencies, the density of states at band edges, and dispersion relations. Another famous example is found in the Anderson localization in photonics, condensed-matter physics, and acoustics [70]. According to scaling theory [71], only 3D disordered materials support metal-insulator transitions, exhibiting dimensionality-dependent transport and localization phenomena. Such an impact of dimensionality on photonics has also recently stimulated the field of synthetic dimensions [72], achieving the observation of higher-dimensional (>3D) phenomena in experimentally valid lower-dimensional systems.

Various high-level photonic platforms and devices are constituted with the combination of different dimensional structures. For example, high-level systems such as signal processors, photovoltaic platforms, and multilevel sensing devices may include 2D receivers and emitters, 1D waveguides or transmission lines, 0D dots or scatterers, and 3D bulk media. In terms of preserving information and energy in high-level systems, lossless information transfer between different dimensional structures is desired. However, this goal is a challenging issue because the forms of eigenmodes and the distributions of eigenvalues strongly depend on the geometrical dimension.

When we consider the electromagnetic boundary condition and the mathematical form of Green’s functions [73], it is straightforward that the continuity of the eigenmodes in different dimensions cannot be satisfied without scattering. Therefore, interdimensional wave transport without information loss requires a methodology based on eigenspectra. In this context, we can envisage the construction of isospectral partner systems in different dimensions—which we call “interdimensional isospectrality”—and their weak coupling between the bound states of each partner system. According to coupled mode theory [39], when the information is composed of the superposition of the bound states of each isospectral partner system, in principle, we can achieve perfect transport of the information between the partner systems. Therefore, the development of isospectral transformation across dimensionality will provide a novel toolkit for signal transport inside a high-level system with mixed dimensions.

Notably, weakly coupled systems in wave mechanics can be interpreted as “wave networks” [74], [75], [76] by assigning wave elements as “network nodes” and the interactions between elements as “network links”. While this network-based viewpoint assigns higher-dimensional systems as high-degree graph networks, the viewpoint allows for the graph description of the Hamiltonian in its matrix form, paving the way for the application of isospectral transformations intensively studied in network science [77]. In this review, we introduce two representative techniques: Householder and Lanczos transformations.

### 4.1 Householder transformation

Because higher-dimensional systems correspond to networks with larger degrees (or a larger number of links for each node) [74], the goal of interdimensional isospectrality is to achieve isospectrality between networks with different degrees. In terms of the matrix formulation of the Hamiltonian, this process handles the number and position of off-diagonal terms of the Hamiltonian matrix. One of the well-known techniques for this process is “tridiagonalization”, transforming a matrix to a tridiagonal form, which corresponds to the nearest-neighbor coupling between elements.

**Figure 5:**
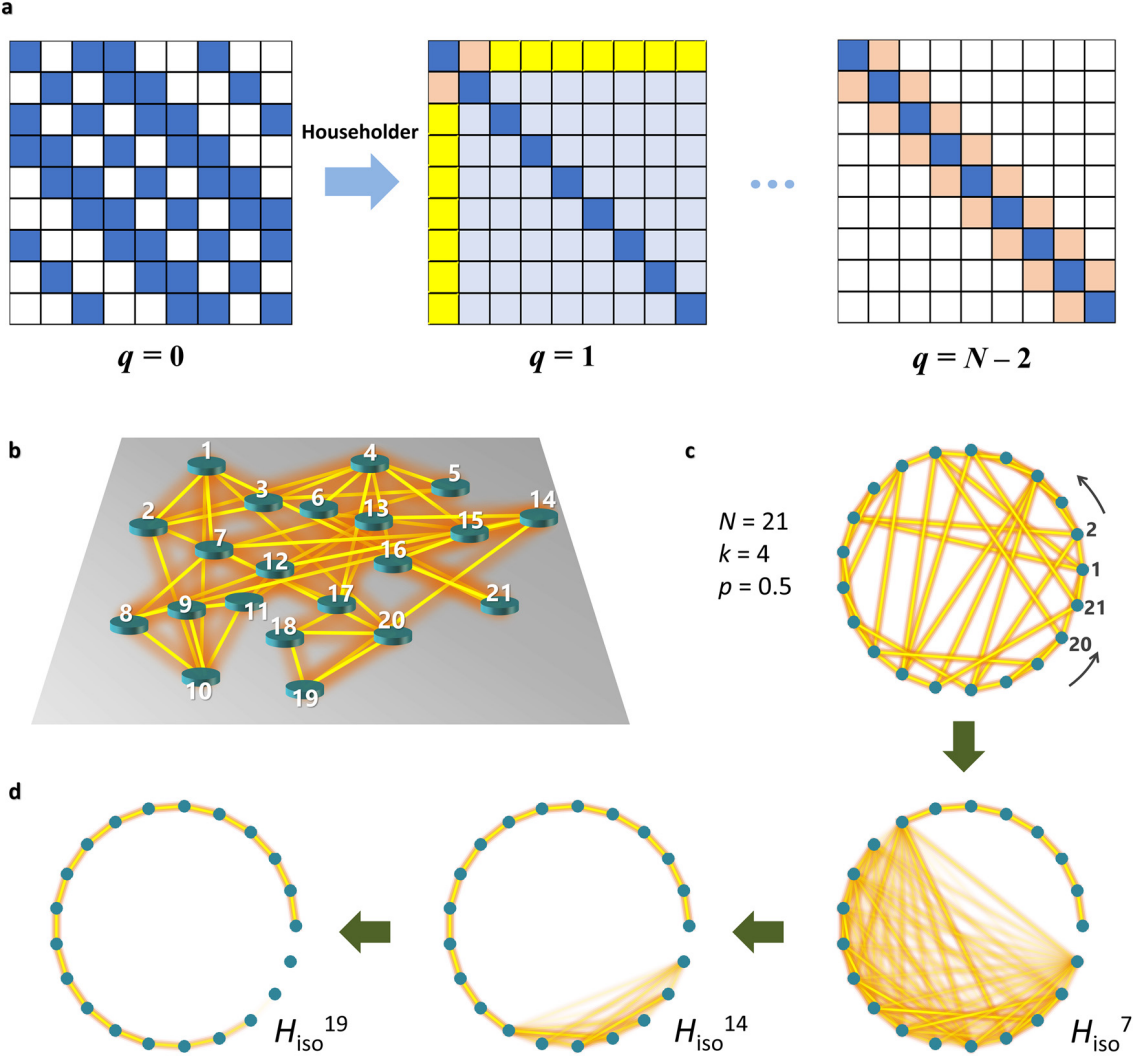
Isospectrality using Householder transformation. (a) A schematic of the Householder-based tridiagonalization for *q* = 0, *q* = 1 and *q* = *N* − 2. Blue and white boxes represent off-diagonal components and zero in the matrices. Reddish boxes denote the calculated first off-diagonal elements. Yellow and light blue boxes denote the removal and alteration of off-diagonal components through each Householder transformation, respectively. (b–d) Householder transformation of a complex network to the isospectral 1D system [74]. (b) A schematic of a hypothetical optical network composed of coupled elements (green cylinders for unit elements and yellow lines for inter-elemental interactions, element number *N* = 21, averaged interaction number per node *k* = 4, and rewiring probability for the Watts–Strogatz process *p* = 0.5). (c) The graph representation of the structure in (b). (d) The resulting nearest-neighbor-coupling network through the sequential Householder transformation. Panels (b)–(d) are adapted with permission from [74], ⓒ Optica.

The Householder transformation is one of the mathematical techniques for tridiagonalization [74, 78] (Figure 5a). First, let us start from the intertwining relation *AH* = *H*_iso_ *A*. When matrix *A* is an orthogonal matrix with *A* ^T^ *A* = *I*, we can use the transformation of the original eigenvalue problem *H*_o_ *ψ*_o,*m*_ = *γ*_*m*_ *ψ*_o,*m*_ as *AH*_o_ *ψ*_o,*m*_ = *AH*_o_ *A* ^T^(*Aψ*_o,*m*_) = *γ*_*m*_(*Aψ*_o,*m*_). In this formulation, we obtain the isospectral Hamiltonian *H*_iso_ = *AH*_o_ *A* ^T^ that satisfies *AH*_o_ = *H*_iso_ *A*. The goal of the Householder transformation is then to find the mathematical form of *A*, which reduces the off-diagonal terms.

For this purpose, assume the sequential removal of the off-diagonal terms until only tridiagonal components remain. The natural form of the isospectral Hamiltonian for this process is *H*_iso_^*q*^ = (*A*_*q*_ *A*_*q*−1_…*A*_2_ *A*_1_)*H*_o_(*A*_*q*_ *A*_*q*−1_…*A*_2_ *A*_1_)^T,^ where *A*_*q*_ *H*_iso_^*q*−1^(*A*_*q*_)^T^ removes *q* row and column off-diagonal components except for the tridiagonal terms in the lower and upper triangular parts of the matrix *H*_iso_^*q*−1^ (Figure 5a). It is known that *A*_*q*_ for the above process is as follows:

(8) $$A_{q}=\left[\begin{array}{ll} \;\;\;I_{q} & O_{q,N-q}\\ O_{N-q,q} & \;\;L_{N-q} \end{array}\right]\text{.}$$

where *N* is the number of elements, *I*_*q*_ is the *q* × *q* identity matrix, *O*_*r*,*s*_ is the *r* × *s* null matrix, respectively, and *L*_*N*−*q*_ = *I*_*N*−*q*_ − 2*uu* ^T^ is the Householder form with the unit normal vector *u* to the reflection plane for the Householder transformation [74, 78]. It is noted that *H*_iso_^*N*−2,^ through sequential transformations, corresponds to the Hamiltonian for the 1D system, which satisfies the isospectrality with the original Hamiltonian *H*_o_.

Figure 5b–d describes the application of the Householder-based tridiagonalization for designing isospectral systems [74]. When an initial system is a hypothetical complex system composed of 21 elements and 4 interactions randomized through Watts–Strogatz rewiring [79] (Figure 5b), its simplified model is described with a graph of 21 nodes and an average of 4 links per node, as shown in Figure 5c. The application of the Householder-based tridiagonalization eventually leads to the graph including only nearest-neighbor links (Figure 5d), which corresponds to the 1D coupled system.

When we employ the Householder transformation to tridiagonalization and subsequent dimensionality engineering, one of the critical issues is degeneracy in eigenspectra. Regarding the relationship between symmetry, dimension, and degeneracy [80], 1D structures do not support degenerate bound states, while higher-dimensional structures allow for degeneracies determined by their symmetries and dimensions. However, because the Householder transformation preserves the entire (nondegenerate and degenerate) eigenvalues of the original Hamiltonian, the tridiagonalization of higher-dimensional structures with degeneracies unavoidably leads to decoupling inside the transformed 1D system. As a result, the application of the Householder transformation to an ordered structure—crystals, quasicrystals, or ordered graph networks—results in an isospectral partner that is composed of multiple 1D substructures that are decoupled from each other [74]. On the other hand, the spectrum of a higher-dimensional disordered structure can be fully reproduced by using a single 1D structure due to its nondegeneracy [74].

**Figure 6:**
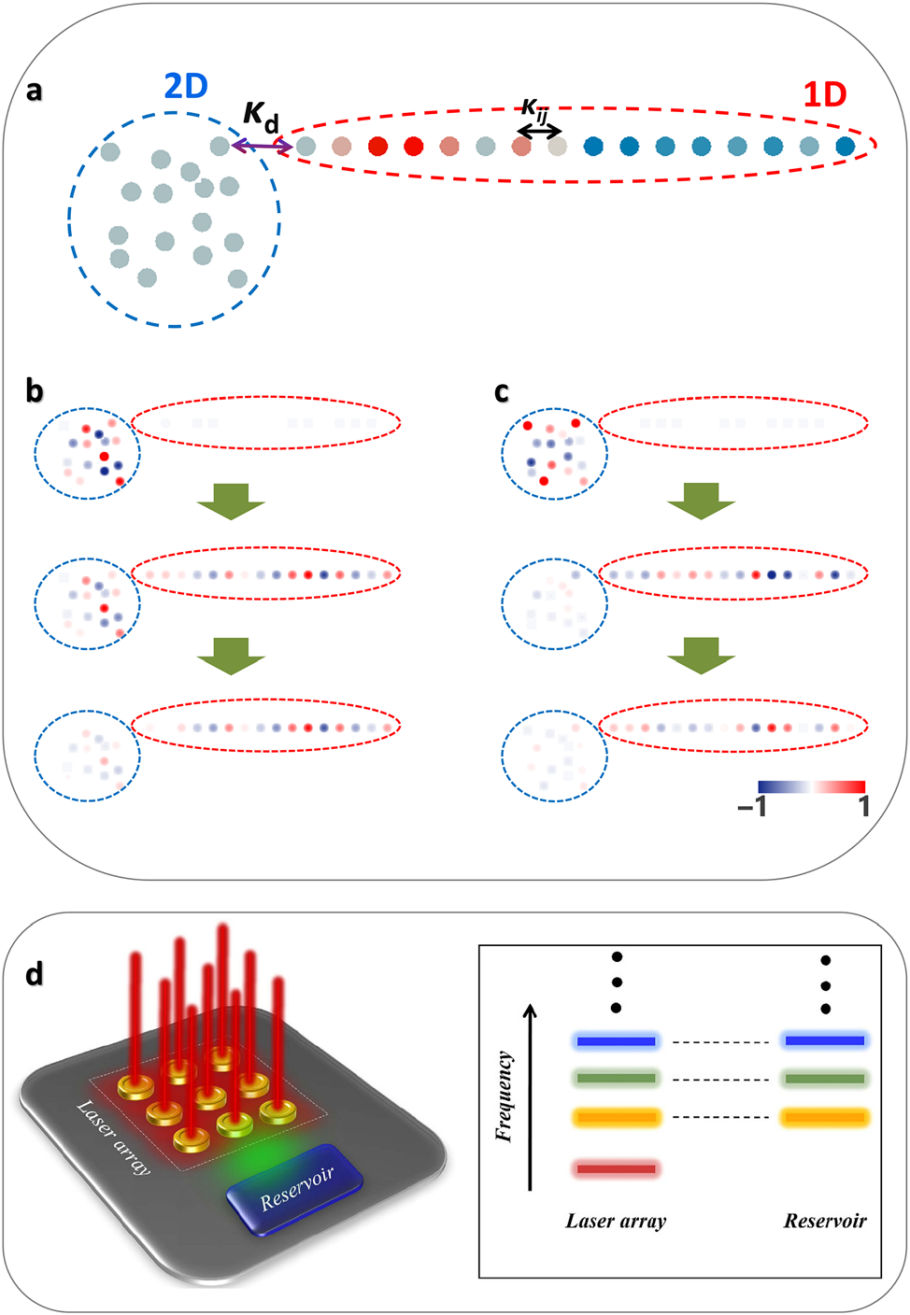
Interdimensional wave transport. (a) A schematic for the interdimensional coupling of 2D and 1D structures [74]. The 1D structure is obtained from the Householder tridiagonalization of the 2D structure. Each atom corresponds to a unit optical element such as an optical waveguide or resonator. *κ*_d_ and *κ*_*ij*_ represent the coupling between isospectral partners and internal coupling, respectively. (b and c) Interdimensional wave transport between the structures for the (b) 3rd and (c) 9th eigenmodes [74]. (d) Single mode two-dimensional laser arrays using SUSY and Householder transformations [81]. A 2D laser array is coupled to an optical reservoir with ground state annihilation. All the eigenfrequencies of both subsystems are matched except for the fundamental one (indicated by the thick red line). By introducing optical coupling between the laser array and the lossy reservoir, only the fundamental lasing mode remains. Panels (a–c) are adapted with permission from [74], ⓒ Optica. Panel (d) is adapted from [81], CC BY 4.0 (https://creativecommons/org/licenses/by/4.0/).

From global phase matching between structures of different dimensions, the application of the Householder transformation enables lossless interdimensional information transport, which is based on the full coupling condition between weakly coupled elements with the same propagation constant or eigenfrequency [39]. For example, consider the coupled system composed of the 2D structure and its 1D partner from the Householder transformation (Figure 6a) [74]. If these substructures are coupled with the coupling strength *κ_d_* much weaker than the internal couplings *κ*_*ij*_ of each structure, we can treat each substructure as a multimodal unit element. Global phase matching then enables the full coupling of every eigenmode between each structure in principle (Figure 6b and c). However, the necessary times for full coupling (or beat length for waveguide elements) between each pair of eigenmodes are different from each other due to distinct effective intermodal coupling.

In addition, the combination of SUSY photonics and Householder transformation provides a novel route to the design of non-Hermitian photonic devices [81]. While the Householder transformation leads to the connection between a higher-dimensional discretized system and its 1D isospectral partner, the SUSY transformation allows ground-state annihilation on such a 1D system. Therefore, at least for weakly coupled, discretized systems, the cascaded application of the Householder and SUSY transformations provides quasi-isospectrality between a higher-dimensional system and the ground-state-annihilated 1D system. This result allows for the interdimensional spectral filtering platform, enabling intriguing applications such as single-mode lasing in a laser array (Figure 6d) [81] and isospectral building blocks for constructing wave networks [64, 82].

### 4.2 Lanczos transformation

In the Householder transformation, the transformation of a higher-dimensional system to the corresponding 1D system does not guarantee a straightforward connection between the nodes (or optical elements) of each system. Therefore, although the spectral information is preserved through the Householder transformation, it is difficult to engineer the profiles of eigenmodes and the following wave dynamics through this transformation. As an advanced method for interdimensional isospectrality, which at least partly enables the engineering of wave dynamics, the use of the specially tailored Lanczos transformation has recently been demonstrated [83].

**Figure 7:**
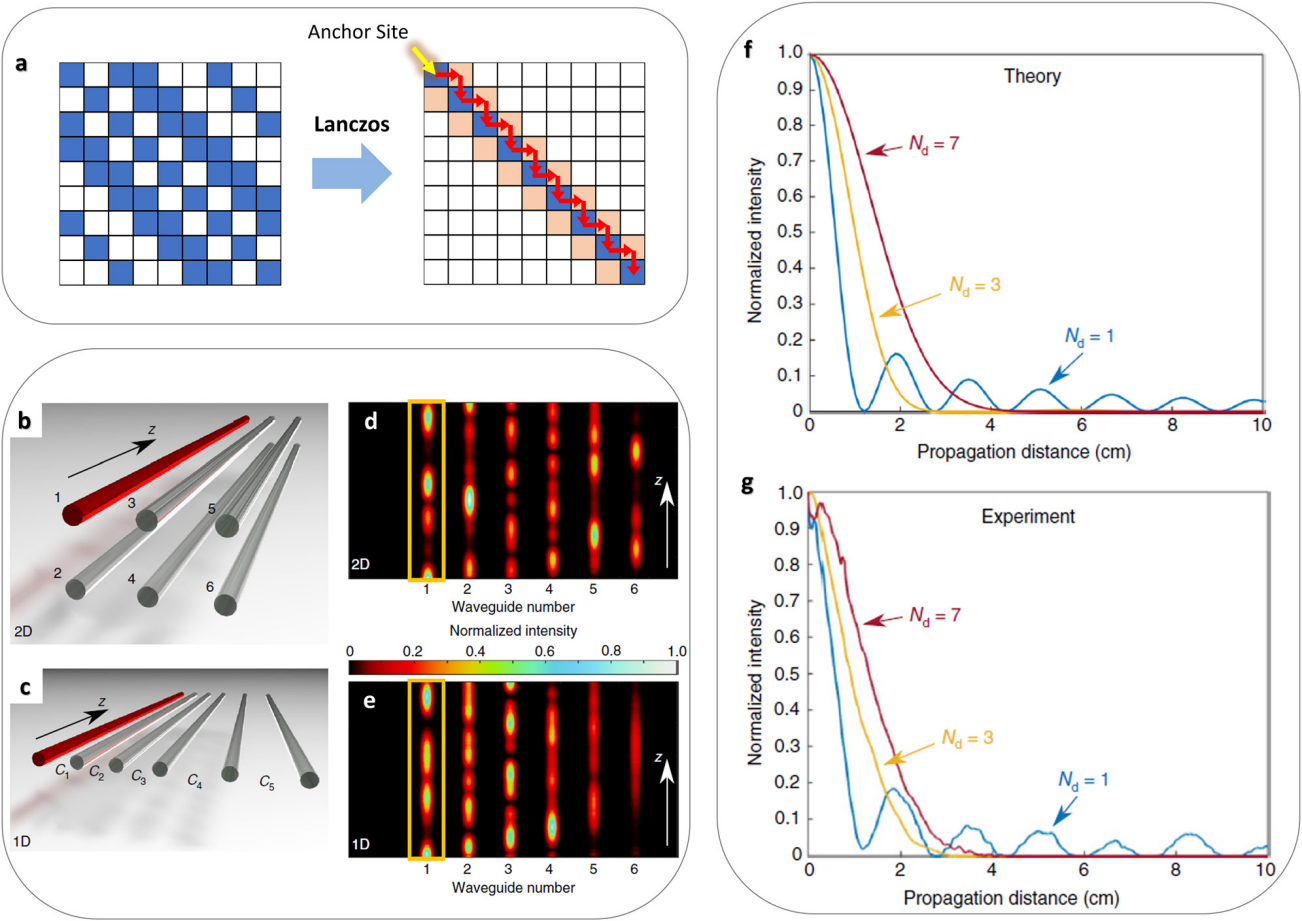
Isospectrality using Lanczos transformation. (a) A schematic of Lanczos-based tridiagonalization from initial 2D to final 1D Hamiltonian matrices [86]. Blue and white represent off-diagonal components and zero in the matrices. Reddish boxes denote the calculated first off-diagonal elements. The yellow arrow depicts the anchor site, and red arrows describe the recursive calculation procedure of Lanczos tridiagonalization. (b and c) The structures connected through the Lanczos transformation [83]: (b) original 2D and (c) transformed 1D coupled waveguide arrays. Red waveguides denote the anchor sites. *C*_*n*_ represents the nonuniform coupling between waveguides, which results from the Lanczos transformation. (d and e) Experimental verification of the reproduced excitation dynamics [83] in the (d) 2D and (e) 1D waveguide arrays. (f) Theoretical and (g) experimental observation of excitation dynamics in the anchor sites of the 1D transformed structures, which are obtained from Lanczos-transformed 1D, 3D and 7D hypercubic lattices [83]. Panel (a) is adapted with permission from [86]. Panels (b–g) are adapted with permission from [83], ⓒ Springer Nature Limited.

The mathematical form of the general Lanczos transformation [84] is similar to that of the Householder transformation as *H*_iso_ = *A* ^†^ *H*_o_ *A*, achieving the tridiagonalization and the corresponding 1D system from the unitary operator *A*. However, in contrast to a series of sub-transformations that remove a part of off-diagonal terms in the Householder transformation (Figure 5a), the Lanczos algorithm is based on the sequential molding of operator *A* from an arbitrary initial column vector with Euclidean norm 1 [84]. In [83], the special form of the Lanczos transformation was suggested by designating the initial column vector to represent one of the elements of the original Hamiltonian *H*_o_, which is called the “anchor site”: e.g., the second element as an anchor site with [0, 1, 0, …]^T^ (Figure 7a). The Lanczos transformation used in the work [83] then preserves the wave component of each normalized eigenmode in this anchor site at every eigenvalue, consequently leading to the corresponding element of the 1D isospectral Hamiltonian *H*_iso_ to the original anchor site. Due to the preserved wave component in the transformed anchor site, wave dynamics around the anchor site is reproduced between different dimensional structures connected through the Lanczos transformation, such as the matching between higher-dimensional corner states and 1D localized states [85].

In [83], with a photonic lattice platform constructed with femtosecond laser writing, isospectrality using Lanczos transformations and wave dynamics with single-site excitation to the anchor site were demonstrated (Figure 7b–e). Figure 7b and c shows the Lanczos transformation between 2D (Figure 7b) and 1D (Figure 7c) waveguide arrays, which possess matched anchor sites (red waveguides). Due to the identical eigenvalues and wave components at the anchor sites of different dimensional structures, the evolution of waves along the anchor waveguide is preserved (yellow boxes in Figure 7d and e), therefore providing the same escape efficiency from the anchor site. This result corresponds to the reproduction of 2D wave dynamics (Figure 7d) in the 1D platform (Figure 7e), at least in the target element of the coupled network.

Beyond the reproduction between 1D and 2D systems, this interdimensional identity of wave dynamics allows access to much higher dimensional (>3D) wave dynamics with a practical 1D platform. Figure 7f and g shows theoretical and experimental verification of higher-dimensional wave diffusion by transforming 3D and 7D structures into their Lanczos counterparts and measuring the escape from the anchor site. In addition to the observation of localization transitions in 3D and 7D wave dynamics in [83], the Lanczos transformation paves the way for the partial reproduction of wave dynamics in higher-dimensional and even complex wave network systems.

## 5 Conclusions and outlook

In the context of independent handling of wave quantities, we have reviewed cornerstones in the field of designing isospectral photonic systems, focusing on the engineering of photonic structures based on analytical methods. The listed works are classified into two categories: SUSY photonics and dimensionality engineering. In both subfields, the methods exploit the intertwining relation *AH* = *H*_iso_ *A*, which is the origin of isospectrality. According to the mathematical nature of operator *A*, each method involves distinctive properties and phenomena, such as ground-state annihilation [20], perfect interdimensional transport [74], and higher-dimensional physics demonstration [83]. The introduced mathematical tools enable the independent manipulation of a set of eigenvalues while controlling the other eigenvalues or other wave quantities, such as transport and localization [37, 74], the spatial profiles of eigenmodes [35, 37, 83], and amplification and dissipation [41, 65, 81].

In SUSY photonics, the key restriction in designing SUSY partner potentials is the difficulty of handling more practical problems: higher-dimensional systems, subwavelength systems, and the handling of optical vector fields. As described in the previous section, SUSY photonics is fundamentally applicable to 1D systems governed by scalar waves. Although several intriguing mathematical techniques, including the separation of variables and time-dependent Darboux or Moutard transformations, have been intensively studied in combination with dimensionality engineering, these methods are usually restricted to particular shapes of potentials and valid only for scalar waves. Furthermore, because SUSY transformations lead to continuous material values in optical or quantum potentials, a mathematical technique for more practical cases involving discretized material values of optical potentials is highly desired. This research direction can also converge with the original problem of isospectrality, manipulating the homogeneous material boundary for achieving isospectrality or quasi-isospectrality with the annihilation of some states.

In interdimensional isospectrality, one of the key restrictions is its limitation to discretized, weakly coupled systems with optical bound states. Because the method in this field is based on handling the existence of each off-diagonal component in the matrix Hamiltonian, the method for manipulating scattering waves and continuous potential landscapes is still absent in contrast to SUSY photonics. In particular, as shown in the constant off-diagonal components in the finite-different approximation, it is difficult to achieve a continuous 1D isospectral partner as the result of the Householder or Lanczos transformation. Another restriction is the difficulty in achieving non-1D transformed systems. Because previous approaches have utilized the tridiagonalization method, the transformation to higher dimensions, e.g., 3D to 2D, is not straightforward. In this context, devising proper transformation methods toward higher dimensions and continuous potential landscapes would be a future research topic.

Beyond the rigorous analytical methods described in this review, various statistical and numerical assessments have shed light on isospectrality by winding off the nonlinear relationship between wave quantities. The concept of hyperuniformity and its extension to “stealthy” [45, 87], [88], [89], which are based on the suppression of long-range fluctuations, allows for the finding of complete bandgap materials despite their highly disordered compositions [90], [91], [92], [93]. Although the stealthy hyperuniformity itself does not guarantee perfectly identical dispersion bands between a disordered material and a specific crystal or quasicrystal, the precise control of the suppressed long-range fluctuations at least enables the matching of bandgap widths. In this context, hyperuniformity can be considered one of the efforts in statistics and material science to satisfy isospectrality between ordered and disordered materials. The key feature of quasi-isospectrality from hyperuniformity is the realization of isotropic bandgap materials [93], distinct from inherent anisotropy in crystals or quasicrystals. Therefore, the statistical molding of stealthy hyperuniform materials corresponds to the independent control of the directivity of materials while preserving the core spectral response. Recently, works on hyperuniformity have been significantly extended to the design of fully transparent materials [94], the realization of nonlocality in composite materials [95], and the extraction of long-range order in prime numbers [88].

The marvelous ability of deep neural networks (DNNs) [96], [97], [98] will also stimulate the design of isospectral partners. The inverse design techniques between wave properties and materials developed through DNNs allow for the design of optical materials while preserving a set of wave quantities [98]. When setting the target wave quantities as a part of spectral information, the construction of optical materials that satisfy quasi-isospectrality while the other wave quantities are determined by the microstructures of DNNs. This impact of DNNs will also pave the way for the independent control of wave quantities not limited to spectral information.

The study of isospectrality in photonics is still in its infancy due to the insufficient versality of analytical methods for handling more practical problems and general wave properties. However, despite existing challenges in this field, there are myriad opportunities based on traditional theories and extended statistical and numerical approaches, which will provide novel design freedom not only in photonics but also in general wave mechanics [34], mechanical systems [99], non-Euclidean physics [75, 76], wave neural networks [100], [101], [102], and synthetic dimensions [72].
